# Waves Propagating in Nano-Layered Phononic Crystals with Flexoelectricity, Microstructure, and Micro-Inertia Effects

**DOI:** 10.3390/nano12071080

**Published:** 2022-03-25

**Authors:** Jun Zhu, Puying Hu, Yudan Chen, Shaowei Chen, Chuanzeng Zhang, Yanzheng Wang, Dongying Liu

**Affiliations:** 1 College of Mechanical Engineering, Zhejiang University of Technology, Hangzhou 310014, China; zhujun@zjut.edu.cn (J.Z.); hpy970106@163.com (P.H.); cyd_0920@163.com (Y.C.); csw31415926@163.com (S.C.); 2 Department of Civil Engineering, University of Siegen, 57068 Siegen, Germany; c.zhang@uni-siegen.de; 3 School of Civil Engineering, Guangzhou University, Guangzhou 510006, China

**Keywords:** phononic crystal, flexoelectricity, microstructure, micro-inertia effects

## Abstract

The miniaturization of electronic devices is an important trend in the development of modern microelectronics information technology. However, when the size of the component or the material is reduced to the micro/nano scale, some size-dependent effects have to be taken into account. In this paper, the wave propagation in nano phononic crystals is investigated, which may have a potential application in the development of acoustic wave devices in the nanoscale. Based on the electric Gibbs free energy variational principle for nanosized dielectrics, a theoretical framework describing the size-dependent phenomenon was built, and the governing equation as well as the dispersion relation derived; the flexoelectric effect, microstructure, and micro-inertia effects are taken into consideration. To uncover the influence of these three size-dependent effects on the width and midfrequency of the band gaps of the waves propagating in periodically layered structures, some related numerical examples were shown. Comparing the present results with the results obtained with the classical elastic theory, we find that the coupled effects of flexoelectricity, microstructure, and micro-inertia have a significant or even dominant influence on the waves propagating in phononic crystals in the nanoscale. With increase in the size of the phononic crystal, the size effects gradually disappear and the corresponding dispersion curves approach the dispersion curves obtained with the conventional elastic theory, which verify the results obtained in this paper. Thus, when we study the waves propagating in phononic crystals in the micro/nano scale, the flexoelectric, microstructure, and micro-inertia effects should be considered.

## 1. Introduction

Phononic crystals are synthetic functional composite materials with spatial periodicity [1,2], which have received considerable research interest due to their unique acoustic properties, such as band gaps and defect states. Phononic crystals have been widely applied in the fields of vibration and noise reduction [3,4], and wave control [5], among others. Considering the convenience of fabrication and testing in early experimental studies [6,7], the structure size of phononic crystals was usually macro scale, which mainly acted on the acoustic wave or ultrasonic wave in the sub-MHz frequency range, and its application was usually limited to filtering [8,9], frequency-dependent guiding [10,11], and wave focusing [12].

With rapid developments in information technology, the working frequencies of many acoustic wave devices have reached GHz or THz, and the size of their components has been reduced to the nanoscale. The phononic crystals in the micro/nano scale many have excellent properties, which might be widely used in the field of high-precision acoustics [13,14].

As known, once the size of a structure reaches the nanoscale, a size-dependent effect has to be taken into account. Flexoelectricity, a kind of electromechanical coupling, describes the spontaneous electrical polarization induced by a nonuniform strain (strain gradient) in ferroelectrics, which have been widely utilized in actuators, sensors, and memory storage, among others [15,16,17]. Unlike piezoelectricity, where a traditional electromechanical effect only exists in non-centrosymmetric materials with no inversion symmetry, the flexoelectric effect does not have this requirement, and can emerge even in centrosymmetric crystals. Such an effect demonstrates strong size-dependence and usually cannot be ignored at the nanoscale. In order to characterize such size effects of the mechanical properties of micro/nano mechanisms, scholars have proposed several theories, which include atomic-scale flexoelectricity based on first principles, lattice dynamics [18], and the phenomenological theory of flexoelectricity based on free energy and constitutive equation of continuum medium [19].

The theoretical description of flexoelectricity in solids dates back to 1964 when Kogan [20] proposed a phenomenological model and theoretically estimated the range of the flexoelectric coupling coefficient. A combination of the phenomenological and microscopic approaches to describe flexoelectricity was presented by Tagantsev [21] in 1980, demonstrating that the flexoelectric effect was accompanied by the non-trivial surface effect and the dynamic effect. An extraordinary achievement in this field was due to the work of Shen and Hu [22,23,24], who established the theoretical framework of continuum mechanics of nano dielectrics by taking into account the flexoelectric effect, the electrostatic force, and the surface effect. Later, an extension of this static continuum theory was carried out by Majdoub et al. [25] based on kinetic energy and the Hamiltonian principle. Based on the framework of the continuum theory proposed by Shen and Hu, Yan et al. [26,27,28] systematically investigated the influence of the flexoelectric effect on the electromechanical coupling response of piezoelectric nanostructures.

Huge effort has also been directed to explore the structural dynamic behaviors of structures in the micro/nano scale by considering the flexoelectric effect. Hu et al. [29,30] studied the propagation of longitudinal waves in elastic dielectrics with the consideration of flexoelectricity, micro-inertia, and strain gradient. Analytical solutions for dispersion relations, phase velocity, and group velocity were calculated; a comparison with the results obtained by the classical elastic theory indicated that the phase velocity and the group velocity were not constant and both varied with the wave number. The Lamb waves propagating in the infinite piezoelectric nanoplates with flexoelectricity and strain gradient elasticity were investigated by Yang et al. [31]. The results show that flexoelectricity could predict a smaller phase velocity, while strain gradient elasticity could predict a greater phase velocity in comparison with the phase velocity of classical piezoelectric Lamb waves.

The influence of the flexoelectric effect on wave propagation in phononic crystals in the nanoscale has also attracted much attention. Based on the constitutive relation of nonlocal theory, Zheng et al. [32] studied the anti-plane and the in-plane elastic waves propagating in 2D nano phononic crystals. Liu et al. [33] pointed out that the flexoelectric effect has a significant influence on the band structure of the nano periodic structure. The influences of flexoelectricity on wave propagation in the two-layered and three-layered phononic crystals are presented by Yang et al. [34]. The results show that the dispersion curves can be possibly modified by flexoelectricity, emphasizing the indispensability of flexoelectricity in the analysis of nanoscale phononic crystals. Besides flexoelectricity, the microstructure and micro-inertia effects have also received a lot of research interest, both of which are also induced due to the size effect. The propagation of Rayleigh waves in a semi-infinite flexoelectric dielectric and a homogeneous centrosymmetric flexoelectric half-space were studied by Qi and Yang et al. [35,36], respectively. It is shown that phase velocity highly depends on both the flexoelectric coefficients and micro-inertia effects. Specifically, they make the phase velocity smaller. In addition, Georgiadis et al. [37] pointed out that the material microstructure effect cannot be negligible, either when the device is reduced to the nanoscale. Furthermore, for high frequency waves, micro-inertia effects also play a very important role since their wavelengths are comparable to the material characteristic length [38]. Thus, both the microstructure and the micro-inertia effects have significant influences on the waves propagating in flexoelectric solids [29]. However, to the authors’ knowledge, little attention has been paid to the waves propagating in nano periodic structures by considering the coupling of the flexoelectric effect, the microstructure, and micro-inertia effects. Thus, the objective of this paper was to analytically investigate the waves propagating in one-dimensional nano phononic crystals based on the flexoelectric theory, incorporating the flexoelectric effect, microstructure, and micro-inertia effects.

## 2. Materials and Methods

**Figure 1:**
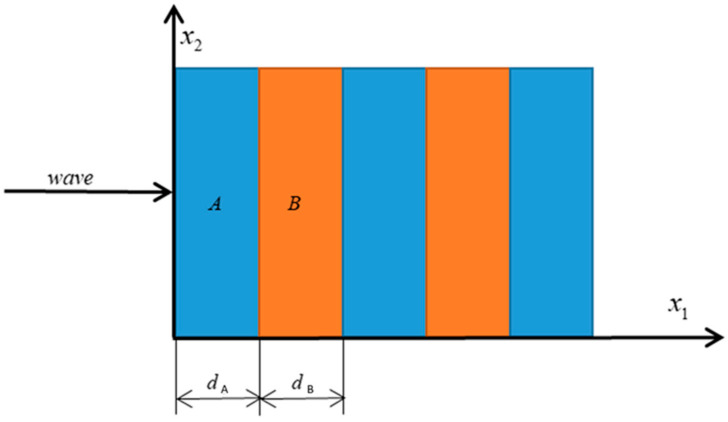
Schematic representation of a one-dimensional layered phononic crystal.

In Figure 1, a layered phononic crystal arranged periodically in the $x_{1}$-direction is shown, which consists of two materials represented by A and B, respectively. The thickness of the unit-cell is $d=d_{A}+d_{B}$, where $d_{A}$ and $d_{B}$ represent the thickness of two-phase materials.

In order to highlight the flexoelectric effect, the microstructure, and micro-inertia effects, centrosymmetric materials are analyzed in this paper. It should be noted that, in order to make the present work more focused, the electromagnetic wave accompanying the electric field is not considered [29,30]. Extending the linear dielectric theory by adding the strain gradient and the electric field terms, the general expression of the electric Gibbs free energy density function can be written as [22]:

(1) $$\begin{array}{c} U=-\frac{1}{2}a_{kl}E_{k}E_{l}+\frac{1}{2}c_{ijkl}\varepsilon_{ij}\varepsilon_{kl}-d_{ijkl}\varepsilon_{ij}V_{kl}+f_{ijkl}E_{i}w_{jkl}\\ +\frac{1}{2}g_{ijklmn}w_{ijk}w_{lmn} \end{array}$$

where $a_{kl}$ is the second-order permittivity tensor, $c_{ijkl}$ is the elastic constant tensor, $\varepsilon_{ij}$ is the strain tensor, $d_{ijkl}$ is the inverse flexoelectric tensor considering the coupling effect of electric field and strain, $f_{ijkl}$ is the flexoelectric coefficient tensor and usually equals to $-d_{ijkl}$ [39], and $g_{ijklmn}$ is approximated by $l_{1}^{2}\delta_{kn}c_{ijlm}$, where $l_{1}$ is the internal characteristic length of the microstructure [40]. $E_{i}$ is the electric field vector formulated in terms of the electric potential ϕ, $w_{jkl}$ is the strain gradient, and $V_{ij}$ is the electric field gradient, which are defined as:

(2) $$E_{i}=-\phi_{,i}\;\;\;\;\;\;\;\;w_{jkl}=\varepsilon_{jk,l}\;\;\;\;\;\;\;\;V_{ij}=E_{i,j}$$

where $\varepsilon_{ij}=\varepsilon_{ji}$, $w_{jkl}=w_{kjl}$, $V_{ij}=V_{ji}$.

Under the assumption of infinitesimal deformation, the constitutive equations of a centrosymmetric medium can be expressed by the Gibbs free energy density function as:

(3) $$\sigma_{ij}=\frac{\partial U}{\partial \varepsilon_{ij}}=c_{ijkl}\varepsilon_{kl}-d_{ijkl}V_{kl}$$

(4) $$\sigma_{ijm}=\frac{\partial U}{\partial w_{ijm}}=-f_{ijkm}E_{k}+l_{1}^{2}\delta_{mk}c_{ijrs}w_{rsk}$$

(5) $$D_{i}=-\frac{\partial U}{\partial E_{i}}=a_{ij}E_{j}+f_{ijkl}w_{jkl}$$

(6) $$Q_{ij}=-\frac{\partial U}{\partial V_{ij}}=d_{klij}\varepsilon_{kl}$$

where $\sigma_{ij}$ is the stress tensor, $D_{k}$ is the electric displacement, $\sigma_{ijm}$ is the higher-order stress tensor, $Q_{ij}$ is the electric quadrupole tensor, and $\sigma_{ij}=\sigma_{ji},\sigma_{ijm}=\sigma_{jim},Q_{ij}=Q_{ji}$.

Based on the variational derivation, the governing equations [21,25] can be derived as:

(7) $${\left(\sigma_{ij}-\sigma_{ijm,m}\right)}_{,j}=\rho {\ddot{u}}_{i}-\frac{\rho l_{2}^{2}}{3}{\ddot{u}}_{i,jj}$$

(8) $${\left(D_{i}-Q_{ij,j}\right)}_{,i}=0$$

where ρ is the mass density and $l_{2}$ is the micro-inertia characteristic length. The resultant quantities

(9) $$P_{i}=\left(\sigma_{ij}-\sigma_{ijm,m}\right)n_{j}+\left(\Delta_{l}n_{l}\right)\sigma_{ijm}n_{m}n_{j}-\Delta_{j}\left(\sigma_{ijm}n_{m}\right)+\frac{\rho l_{2}^{2}}{3}n_{j}{\ddot{u}}_{i,j}$$

(10) $$R_{i}=\sigma_{ijm}n_{j}n_{m}$$

(11) $$q\;=\;\left(D_{i}-Q_{ij,j}\right)n_{i}\;+\;\left(\Delta_{l}n_{l}\right)Q_{ij}n_{i}n_{j}\;+\;\Delta_{i}\left(Q_{ij}n_{j}\right)$$

(12) $$r=Q_{ij}n_{i}n_{j}$$

should be continuous at the interface of the two materials, where $n_{i}$ is the unit normal vector of the solid boundary. $\Delta_{i}\;\equiv \;\left(\delta_{ij}-n_{i}n_{j}\right)\partial_{j}$, $\Delta \;\equiv \;n_{i}\partial_{i}$ and $\partial_{i}$ represent the partial derivative with respect to $x_{i}$.

In this paper, the longitudinal waves propagating along the $x_{1}$ direction in the layered phononic crystal are investigated. Substituting the constitutive relation represented by the non-zero displacement component $u_{1}$ and the potential ϕ into Equations (7) and (8), the governing equations can be reduced to:

(13) $$c_{11}\frac{\partial^{2}u_{1}}{\partial x_{1}^{2}}+d_{11}\frac{\partial^{3}\phi }{\partial x_{1}^{3}}-f_{11}\frac{\partial^{3}\phi }{\partial x_{1}^{3}}-l_{1}^{2}c_{11}\frac{\partial^{4}u_{1}}{\partial x_{1}^{4}}=\rho \frac{\partial^{2}u_{1}}{\partial t^{2}}-\frac{1}{3}\rho l_{2}{}^{2}\frac{\partial^{4}u_{1}}{\partial x_{1}^{2}\partial t^{2}}$$

(14) $$-a_{11}\frac{\partial^{2}\phi }{\partial x_{1}^{2}}+f_{11}\frac{\partial^{3}u_{1}}{\partial x_{1}^{3}}-d_{11}\frac{\partial^{3}u_{1}}{\partial x_{1}^{3}}=0$$

The solution of the above equations can be provided by the following form:

(15) $$\phi \left(x_{1},t\right)=A\exp(i\alpha x_{1})\exp(-i\omega t)$$

(16) $$u_{1}\left(x_{1},t\right)=\beta A\exp(i\alpha x_{1})\exp(-i\omega t)$$

where A is the undetermined coefficient, β is the amplitude ratio, $i=\sqrt{-1}$ is the imaginary unit, α is the wave number, ω is the angular frequency. By substituting Equations (15) and (16) into Equations (13) and (14), we get:

(17) $$-c_{11}\alpha^{2}\beta A-d_{11}i\alpha^{3}A+f_{11}i\alpha^{3}A-l_{1}^{2}c_{11}\alpha^{4}\beta A+\omega^{2}\rho \beta A+\frac{1}{3}\rho l_{2}{}^{2}\alpha^{2}\omega^{2}\beta A=0$$

(18) $$a_{11}\alpha^{2}A-f_{11}i\alpha^{3}\beta A+d_{11}i\alpha^{3}\beta A=0$$

which can be further written in the matrix form as

(19) $$\left[\begin{array}{cc} -c_{11}\alpha^{2}-l_{1}^{2}c_{11}\alpha^{4}+\omega^{2}\rho +\frac{\rho l_{2}{}^{2}\alpha^{2}\omega^{2}}{3} & 2f_{11}i\alpha^{3}\\ -2f_{11}i\alpha^{3} & a_{11}\alpha^{2} \end{array}\right]\left[\begin{array}{c} \beta A\\ A \end{array}\right]=0$$

in which the relationship $f_{11}=-d_{11}$ is adopted. In order to obtain the nontrivial solution of A, the determinant value of the coefficient matrix must be zero, which leads to:

(20) $$\alpha^{2}\left(c_{11}a_{11}\alpha^{2}+l_{1}^{2}c_{11}a_{11}\alpha^{4}-\omega^{2}\rho a_{11}-\frac{\rho l_{2}{}^{2}a_{11}\alpha^{2}\omega^{2}}{3}+4f_{11}{}^{2}\alpha^{4}\right)=0$$

Six roots of $\alpha_{m}$ (m=1~6) can be obtained by solving the polynomial equation, which has the form of $\alpha_{1}=-\alpha_{2}$, $\alpha_{3}=-\alpha_{4}$, $\alpha_{5}=-\alpha_{6}=0$. Then, the solutions of the displacement and the electric potential can be expressed as:

(21) $$\begin{array}{c} \phi \left(x_{1},t\right)=\left[A_{1}\exp(i\alpha_{1}x_{1}\right)+A_{2}\exp(i\alpha_{2}x_{1})+A_{3}\exp(i\alpha_{3}x_{1})\\ +A_{4}\exp(i\alpha_{4}x_{1})+A_{5}x_{1}+A_{6}]\exp(-i\omega t) \end{array}$$

(22) $$\begin{array}{c} u_{1}\left(x_{1},t\right)=\left[\beta_{1}A_{1}\exp(i\alpha_{1}x_{1}\right)+\beta_{2}A_{2}\exp(i\alpha_{2}x_{1})+\beta_{3}A_{3}\exp(i\alpha_{3}x_{1})\\ +\beta_{4}A_{4}\exp(i\alpha_{4}x_{1})]\exp(-i\omega t) \end{array}$$

where $A_{m}$(m=1~6) are the undetermined coefficients; the four roots $\alpha_{m}$ and the corresponding amplitude ratios $\beta_{m}$ (m=1~4) have the following form:

(23) $$\alpha_{1}=-\alpha_{2}=\sqrt{\frac{-\left(c_{11}a_{11}-\rho l_{2}{}^{2}a_{11}\omega^{2}/3\right)+\sqrt{{\left(c_{11}a_{11}-\rho l_{2}{}^{2}a_{11}\omega^{2}/3\right)}^{2}+4\omega^{2}\rho a_{11}\left(4f_{11}{}^{2}+l_{1}^{2}c_{11}a_{11}\right)}}{2\left(4f_{11}{}^{2}+l_{1}^{2}c_{11}a_{11}\right)}}\;\alpha_{3}=-\alpha_{4}=\sqrt{\frac{-\left(c_{11}a_{11}-\rho l_{2}{}^{2}a_{11}\omega^{2}/3\right)-\sqrt{{\left(c_{11}a_{11}-\rho l_{2}{}^{2}a_{11}\omega^{2}/3\right)}^{2}+4\omega^{2}\rho a_{11}\left(4f_{11}{}^{2}+l_{1}^{2}c_{11}a_{11}\right)}}{2\left(4f_{11}{}^{2}+l_{1}^{2}c_{11}a_{11}\right)}}$$

(24) $$\beta_{m}=\frac{2f_{11}i\alpha_{m}^{3}}{c_{11}\alpha_{m}^{2}+l_{1}^{2}c_{11}\alpha_{m}^{4}-\omega^{2}\rho -\rho l_{2}{}^{2}\alpha_{m}^{2}\omega^{2}/3}\;\left(m=1\sim 4\right)$$

Here, the transfer matrix method was employed to calculate the band structure of the layered phononic crystals. In virtue of the relationship between the state vectors of each layer and the Bloch theorem, the dispersion or characteristic equation can be obtained. In addition, the transmission coefficient describing the transmission behavior of waves in finite periodic laminates can also be calculated by this method. Here, $v={\left(\varphi ,u_{1},P_{1},R_{1},q,r\right)}^{T}$ is selected as the state vector, and the expressions of the components, $P_{1},R_{1},q,r$ can be written as:

(25) $$P_{1}=\overset{4}{\underset{m=1}{\sum }}A_{m}(c_{11}\beta_{m}i\alpha_{m}+2f_{11}\alpha_{m}^{2}+l_{1}^{2}c_{11}\beta_{m}i\alpha_{m}^{3}-\frac{1}{3}\rho l_{2}{}^{2}\beta_{m}\omega^{2}i\alpha_{m})e^{i\alpha_{m}x_{1}}e^{-i\omega t}\;\begin{array}{c} R_{1}=\left(\overset{4}{\underset{m=1}{\sum }}A_{m}\left(f_{11}i\alpha_{m}-l_{1}^{2}c_{11}\beta_{m}\alpha_{m}^{2}\right)e^{i\alpha_{m}x_{1}}+A_{5}f_{11}\right)e^{-i\omega t} \end{array}\;q=(\overset{4}{\underset{m=1}{\sum }}A_{m}\left(-a_{11}i\alpha_{m}-2f_{11}\beta_{m}\alpha_{m}^{2}\right)e^{i\alpha_{m}x_{1}}-A_{5}a_{11})e^{-i\omega t}\;r=\overset{4}{\underset{m=1}{\sum }}-f_{11}A_{m}i\beta_{m}\alpha_{m}e^{i\alpha_{m}x_{1}}e^{-i\omega t}$$

Taking the *m*-th unit-cell for example, the state vectors in each sublayer can be written in the matrix form as:

(26) $$v_{j}^{\left(m\right)}=M_{j}\left(x_{1}\right){\left\{A_{1},A_{2},A_{3},A_{4},A_{5},A_{6}\right\}}_{j}^{T}$$

in which $M_{j}\left(x_{1}\right)$ is the state matrix and the subscript $j=1,\begin{array}{c} 2 \end{array}$ represents the first and second sublayers of the unit-cell, respectively. The expression of the state matrix $M_{j}\left(x_{1}\right)$ can be represented by:

(27) $$M_{j}\left(x_{1}\right)=\left[\begin{array}{c} e^{i\alpha_{1}x_{1}}\\ \beta_{1}e^{i\alpha_{1}x_{1}}\\ \left[c_{11}\beta_{1}i\alpha_{1}+f_{11}\alpha_{1}^{2}+l_{1}^{2}c_{11}\beta_{1}i\alpha_{1}^{3}-\frac{1}{3}\rho l_{2}^{2}\beta_{1}\omega^{2}i\alpha_{1}\right]e^{i\alpha_{1}x_{1}}\\ \left[f_{11}i\alpha_{1}-l_{1}^{2}c_{11}\beta_{1}\alpha_{1}^{2}\right]e^{i\alpha_{1}x_{1}}\\ \left[-\varepsilon_{11}i\alpha_{1}-f_{11}\beta_{1}\alpha_{1}^{2}\right]e^{i\alpha_{1}x_{1}}\\ i\beta_{1}\alpha_{1}e^{i\alpha_{1}x_{1}} \end{array}\right.\begin{array}{c} e^{i\alpha_{2}x_{1}}\\ \beta_{2}e^{i\alpha_{2}x_{1}}\\ \left[c_{11}\beta_{2}i\alpha_{2}+f_{11}\alpha_{2}^{2}+l_{1}^{2}c_{11}\beta_{2}i\alpha_{2}^{3}-\frac{1}{3}\rho l_{2}^{2}\beta_{2}\omega^{2}i\alpha_{2}\right]e^{i\alpha_{2}x_{1}}\\ \left[f_{11}i\alpha_{2}-l_{1}^{2}c_{11}\beta_{2}\alpha_{2}^{2}\right]e^{i\alpha_{2}x_{1}}\\ \left[-\varepsilon_{11}i\alpha_{2}-f_{11}\beta_{2}\alpha_{2}^{2}\right]e^{i\alpha_{2}x_{1}}\\ i\beta_{2}\alpha_{2}e^{i\alpha_{2}x_{1}} \end{array}\;\begin{array}{c} e^{i\alpha_{3}x_{1}}\\ \beta_{3}e^{i\alpha_{3}x_{1}}\\ \left[c_{11}\beta_{3}i\alpha_{3}+f_{11}\alpha_{3}^{2}+l_{1}^{2}c_{11}\beta_{3}i\alpha_{3}^{3}-\frac{1}{3}\rho l_{2}^{2}\beta_{3}\omega^{2}i\alpha_{3}\right]e^{i\alpha_{3}x_{1}}\\ \left[f_{11}i\alpha_{3}-l_{1}^{2}c_{11}\beta_{3}\alpha_{3}^{2}\right]e^{i\alpha_{3}x_{1}}\\ \left[-\varepsilon_{11}i\alpha_{3}-f_{11}\beta_{3}\alpha_{3}^{2}\right]e^{i\alpha_{3}x_{1}}\\ i\beta_{3}\alpha_{3}e^{i\alpha_{3}x_{1}} \end{array}{\left.\begin{array}{ccc} e^{i\alpha_{4}x_{1}} & x_{1} & 1\\ \beta_{4}e^{i\alpha_{4}x_{1}} & 0 & 0\\ \left[c_{11}\beta_{4}i\alpha_{4}+f_{11}\alpha_{4}^{2}+l_{1}^{2}c_{11}\beta_{4}i\alpha_{4}^{3}-\frac{1}{3}\rho l_{2}^{2}\beta_{4}\omega^{2}i\alpha_{4}\right]e^{i\alpha_{4}x_{1}} & 0 & 0\\ \left[f_{11}i\alpha_{4}-l_{1}^{2}c_{11}\beta_{4}\alpha_{4}^{2}\right]e^{i\alpha_{4}x_{1}} & f_{11} & 0\\ \left[-\varepsilon_{11}i\alpha_{4}-f_{11}\beta_{4}\alpha_{4}^{2}\right]e^{i\alpha_{4}x_{1}} & -\varepsilon_{11} & 0\\ i\beta_{4}\alpha_{4}e^{i\alpha_{4}x_{1}} & 0 & 0 \end{array}\right]}_{j}$$

Therefore, in the *m*-th unit-cell, the state vectors on the two surfaces of the first sublayer can be expressed as:

(28) $$v_{1L}^{\left(m\right)}=M_{1}\left(0\right){\left\{A_{1},A_{2},A_{3},A_{4},A_{5},A_{6}\right\}}_{1}^{T}$$

(29) $$v_{1R}^{\left(m\right)}=M_{1}\left(d_{A}\right){\left\{A_{1},A_{2},A_{3},A_{4},A_{5},A_{6}\right\}}_{1}^{T}$$

in which the subscripts *L* and *R* denote the left and right sides of the sublayer, respectively. Similarly, the state vectors on the two sides of the second sublayer can be denoted as:

(30) $$v_{2L}^{\left(m\right)}=M_{2}\left(d_{A}\right){\left\{A_{1},A_{2},A_{3},A_{4},A_{5},A_{6}\right\}}_{2}^{T}$$

(31) $$v_{2R}^{\left(m\right)}=M_{2}\left(d\right){\left\{A_{1},A_{2},A_{3},A_{4},A_{5},A_{6}\right\}}_{2}^{T}$$

Considering the continuity conditions at the interfaces between the two neighboring layers, the relationship between the state vectors on the left and the right sides of the m-th unit-cell can be obtained:

(32) $$v_{1R}^{\left(m\right)}=v_{2L}^{\left(m\right)}$$

(33) $$v_{2R}^{\left(m\right)}=M_{2}\left(d\right)M_{2}{\left(d_{A}\right)}^{-1}M_{1}\left(d_{A}\right)M_{1}{\left(0\right)}^{-1}v_{1L}^{\left(m\right)}$$

For finite laminates composed of N unit-cells, the total transfer matrix M can be given as $M={\left(M_{2}\left(d\right)M_{2}{\left(d_{A}\right)}^{-1}M_{1}\left(d_{A}\right)M_{1}{\left(0\right)}^{-1}\right)}^{N}$, which can be further utilized to investigate the transmission behavior of the waves propagating in the laminates.

On incorporating the well-known Bloch theorem, the dispersion relation of the waves propagating in the infinite periodic laminates can be obtained by making the determinant of the coefficient matrix zero:

(34) $$\left|M-e^{ikd}I\right|=0$$

where I is the identity matrix and k is the Bloch wave number. By calculating all the Bloch wave numbers k and the corresponding ω in the first Brillouin zone $\left[0,\pi /d\right]$, the band gap distribution of the one-dimensional periodic structure can be obtained.

## 3. Results and Discussion

In this section, the waves propagating in the periodic laminates considering the coupling of flexoelectricity, as well as the microstructure and micro-inertia effects are investigated. Centrosymmetric materials are attractive in exploring the flexoelectric phenomenon since there is no piezoelectricity existing in them; therefore, two typical centrosymmetric materials *A* (BaTiO_3_) and *B* (SrTiO_3_) in cubic phase are used in this study. The material parameters are shown in Table 1. The values of $l_{1}$ and $l_{2}$ are related to the underlying micro-structure of the material and usually restricted to the magnitude of nanometers [29,36]. In order to simplify the solution of the problem, we let $l_{1}=l_{2}=l$ in the numerical calculation, and assume that the flexoelectric coefficients $f_{11}$ of the two materials are equal and vary in the range of $[0-2\times 10^{-7}\;C/m]$. In the following figures, ELA, FE, and MME are used to denote the constitutive relations considering pure elasticity, the flexoelectric effect, and the microstructure/micro-inertia effects, respectively.

**Figure 2:**
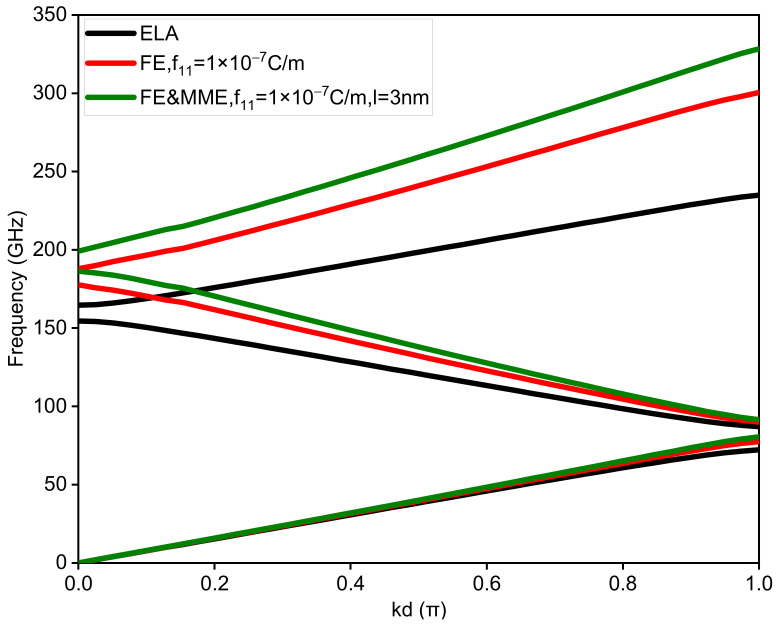
Band structures of the binary phononic crystals considering ELA, FE, and FE and MME, respectively.

Firstly, the band structure of the binary phononic crystals with the sublayers of equal thickness, is studied, i.e., $d_{A}=d_{B}$. Figure 2 illustrates the first two band gaps for the three situations, i.e., ELA, FE, and FE and MME, in which the thickness of each sublayer is set as 20 nm and the Bloch wave number k is normalized by π/d. A comparison between the three cases shows that the size-effects induced the flexoelectricity, and the microstructure and micro-inertia effects have a considerable influence on the dispersion curves, especially the widths and midfrequencies of the band gaps. In addition, it is found that the band gap in the higher frequency region (the second band gap) is more sensitive to the size effects than the band gaps in the low frequency region (the first band gap). The ranges and corresponding midfrequencies of the first two band gaps are given in Table 2.

On comparing the dispersion curve considering only the FE with the dispersion curve considering both the FE and the MME in Figure 2, we find that the influence of the MME on the first band gap is small. However, both the FE and the MME have a significant influence on the second band gaps (see the dispersion curves with the wave number being zero). This identifies if the FE or the FE and MME will greatly increase the midfrequency and the width of the second band gap. The influence of the FE and the MME will become larger with the increase in frequency. Thus, both the FE and the MME cannot be ignored for the analysis of band gaps in a high frequency region.

**Figure 3:**
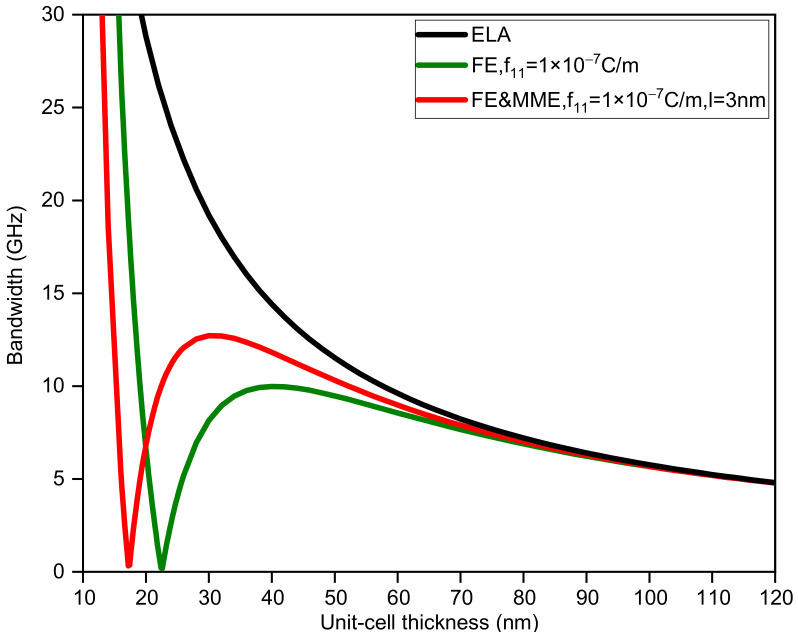
The bandwidth of the first band gap via unit-cell thickness $\left(d_{A}=d_{B}\right)$.

**Figure 4:**
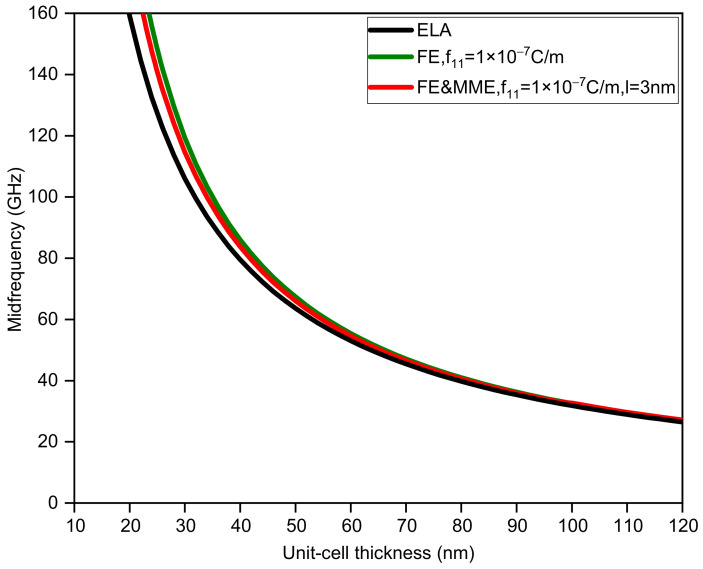
The midfrequency of the first band gap via unit-cell thickness $\left(d_{A}=d_{B}\right)$.

In addition, considering ELA, FE, and FE and MME, we also calculate how the change in unit-cell thickness affects the first band gap. For binary phononic crystals with the same sublayer thickness, the bandwidth and midfrequency changes with respect to the unit-cell thickness are shown in Figure 3 and Figure 4. It is obvious that the large discrepancy between the three curves in Figure 3 mainly occurs when the thickness of the unit cell is small, especially less than 50 nm. If we do not take the FE and the MME into account, the bandwidth monotonically decreases as the thickness of the unit-cell grows. In contrast, the bandwidth variation curves considering the FE and MME behave differently from the purely elastic case, which initially drop from large values to zero, implying the closing of the band gap. Furthermore, the band gaps reopen to maximum width and decrease gradually. However, in all the three cases, midfrequency varies in the same trend to a smaller value as the unit-cell gets thicker in which FE and the MME predict higher midfrequency for the same thickness. It is evident that when the unit-cell thickness is relatively large, the size effects (i.e., the FE and the MME) disappear; see the three overlapping lines in Figure 3 and Figure 4, which can sort of prove the reasonability of the results in this paper.

**Figure 5:**
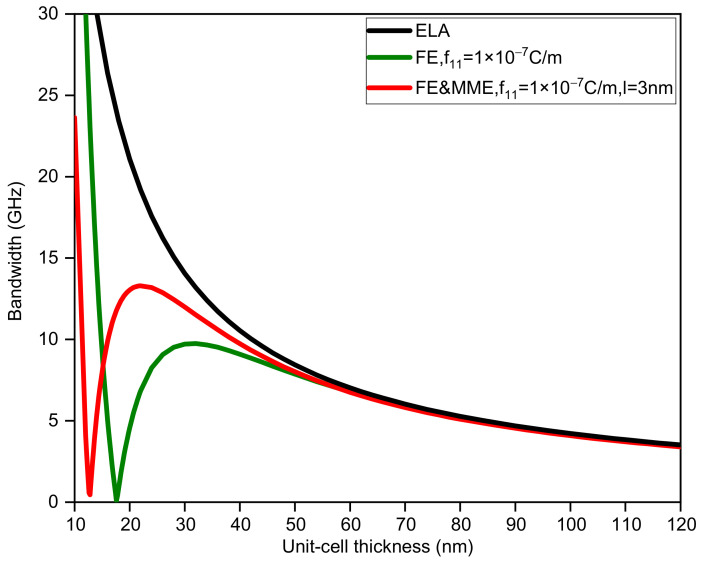
The bandwidth of the first band gap via unit-cell thickness ($d_{A}=2d_{B}$).

**Figure 6:**
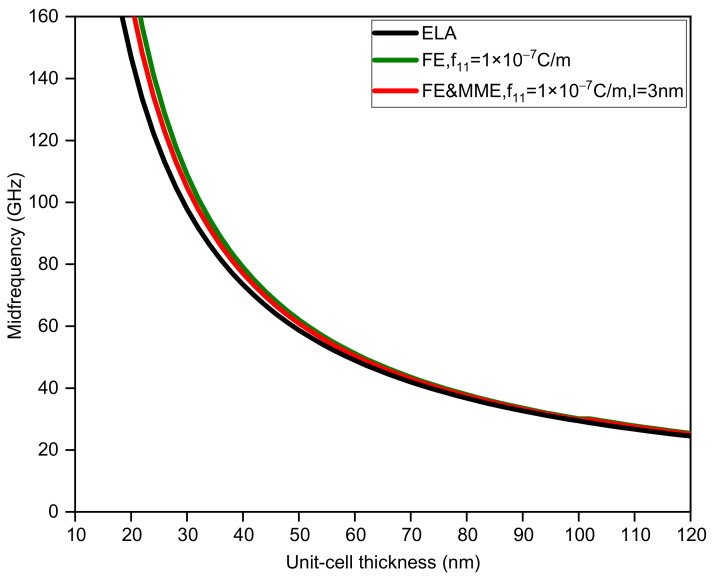
The midfrequency of the first band gap via unit-cell thickness ($d_{A}=2d_{B}$).

**Figure 7:**
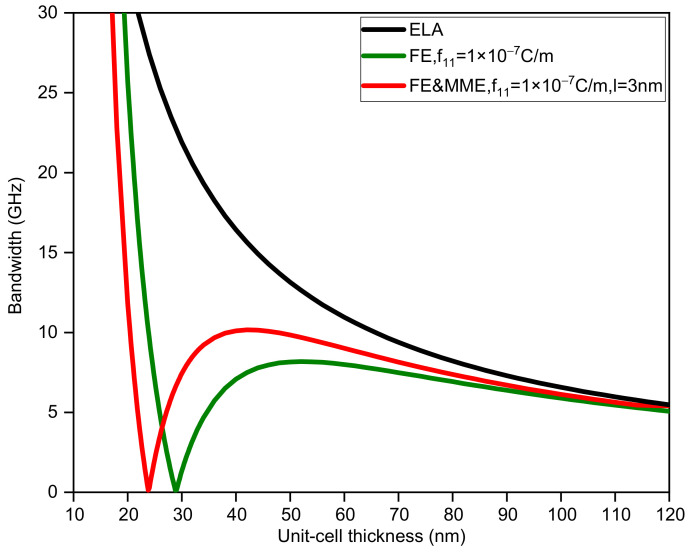
The bandwidth of the first band gap via unit-cell thickness ($d_{A}=0.5d_{B}$).

**Figure 8:**
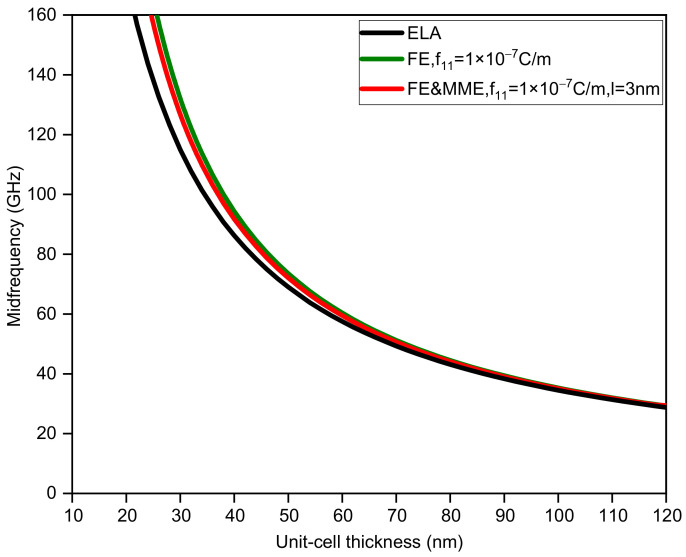
The midfrequency of the first band gap via unit-cell thickness ($d_{A}=0.5d_{B}$).

Figure 5, Figure 6, Figure 7 and Figure 8 display the variations in the bandwidth and midfrequency of the first band gap for the three situations, where the sub-layer thickness is no longer equal and two other thickness ratios ($d_{A}=2d_{B},\;d_{A}=0.5d_{B}$) are adopted. It can be observed that the FE and MME have similar effects on the bandwidth of the band gap as well as the midfrequency in the current calculation range. However, it can be found that the critical length, where the size effect disappears, relies on not only the thickness of the unit cell, but also the thickness ratio of the two phases. A larger thickness ratio of phase A results in the closing of the band gap at smaller unit-cell thickness, while reducing the midfrequency of the first band gap. On the other hand, such an increase in phase A would also speed up the curves, considering size-dependent effects approaching the elastic results, which is easier to observe from a comparison of the bandwidth variation curves.

**Figure 9:**
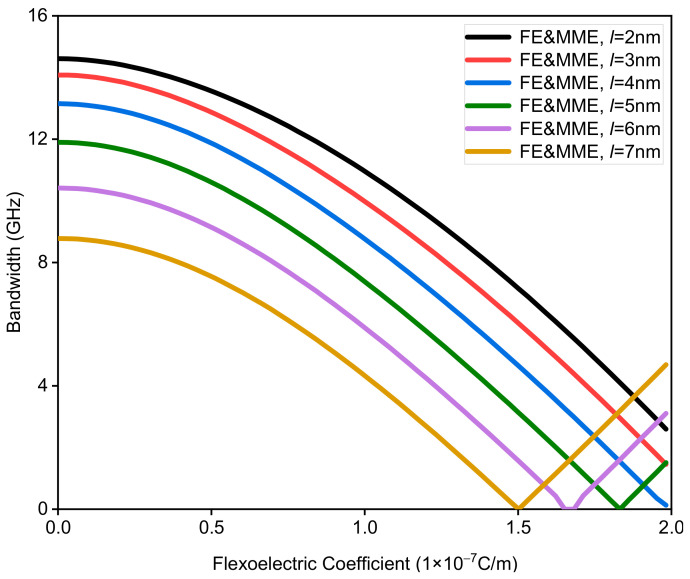
The bandwidth of the first band gap via the flexoelectric coefficient of a phononic crystal.

**Figure 10:**
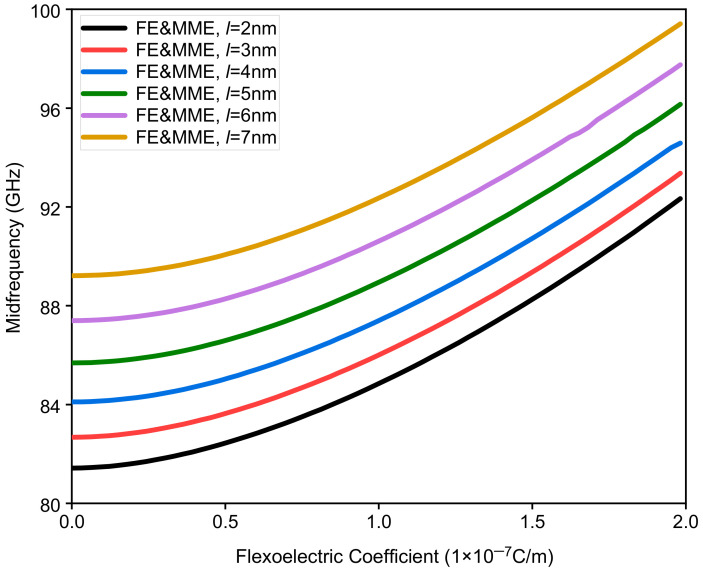
The midfrequency of the first band gap via the flexoelectric coefficient of a phononic crystal.

This part mainly studies the influence of different flexoelectric coefficients on band gap characteristics, considering the microstructure and micro-inertia effects. Assuming that the thickness of each sub-layer is 20 nm, the variation range of the microstructure and micro-inertia characteristic length is set as 2 nm to 7 nm, and the flexoelectric coefficient is within the range of 0 to 2 × 10^−7^ C/m. The variations in the bandwidth of the first band gap with different flexoelectric coefficients and characteristic lengths are shown in Figure 9. It is interesting to know that the first band gaps with different characteristic lengths gradually close and then reopen as the flexoelectric coefficient increases. Moreover, for the fixed flexoelectric coefficient, the bandwidth decreases with increase in the characteristic length before the closing of the band gap, while the situation is totally the opposite after the reopening of the band gap; see Figure 9. Figure 10 shows the variation in the midfrequency of the first band gaps with the increase in the flexoelectric coefficient for the different characteristic lengths. When the characteristic length is fixed, the midfrequency of the band gap increases monotonously as the flexoelectric coefficient grows. Meanwhile, the bigger characteristic length also gives rise to the increment in the midfrequency for the same flexoelectric coefficient. From the above analysis, we can conclude that the coupling effect of the flexoelectricity, the microstructure, and the micro-inertia cannot be ignored for the dispersion curves of the waves propagating in nano-scaled phononic crystals.

## 4. Conclusions

Based on the transfer matrix method, the waves propagating in the nano-layered phononic crystals are investigated, wherein the flexoelectric effect and the microstructure and micro-inertia effects are considered. To demonstrate the influence of these size-dependent effects, the dispersion curves of periodic structures with flexoelectric effect as well as with microstructure and micro-inertia effects are compared with that obtained by the classical elastic theory. It was found that the discrepancy among the midfrequency and bandwidth of the band gap obtained from three theoretical models becomes more evident as the thickness of unit-cell decreases. Furthermore, the influence of thickness ratio, characteristic length *l*, and flexoelectric coefficient on band gap distribution are subsequently explored through parametric analysis. In summary, it was proved in this paper that the flexoelectric effect, microstructure, and micro-inertia effects are of great significance for the accurate control and practical application of elastic waves propagating in nanoscale devices.

## Figures and Tables

**Table 1:** Material parameters of *A* and *B* [29,34].

*A*	*B*
$\rho =6020\;\mathrm{kg}/m^{3}$	$\rho =5120\;\mathrm{kg}/m^{3}$
$c_{11}=162\times 10^{9}\;N/m^{2}$	$c_{11}=350\times 10^{9}\;N/m^{2}$
$a_{11}=35.4\times 10^{-9}\;F/m$	$a_{11}=2.6\times 10^{-9}\;F/m$

**Table 2:** Ranges and midfrequencies of the band gaps.

		ELA	FE	FE&MME
1st band gap	Range (GHz)	72–87	79–89	80–92
	Midfrequency (GHz)	79.5	84	86
2nd band gap	Range (GHz)	154–164	177–187	186–199
	Midfrequency (GHz)	159	182	192

## Data Availability

Not applicable.
